# Introducing field-programmable gate arrays in genotype phasing and imputation

**DOI:** 10.1093/bioadv/vbae114

**Published:** 2024-07-30

**Authors:** Lars Wienbrandt, David Ellinghaus

**Affiliations:** Institute of Clinical Molecular Biology, Kiel University, Am Botanischen Garten 11, 24108 Kiel, Germany; Institute of Clinical Molecular Biology, Kiel University, Am Botanischen Garten 11, 24108 Kiel, Germany

## Abstract

**Summary:**

We recently developed *EagleImp*, a free software that combines genotype phasing and imputation in a single tool. By introducing algorithmic and technical improvements we accelerated the classical two-step approach using *Eagle2* and *PBWT*. Here, we demonstrate how to use field-programmable gate arrays (FPGAs) to accelerate *EagleImp* even further by a factor of up to 93% without loss of phasing and imputation quality. Due to the speed advantage over a not accelerated processor-based implementation, the FPGA extension of *EagleImp* allows the user to choose a more resource-intensive parameter setting in exchange for computation time to further improve phasing and imputation quality.

**Availability and implementation:**

*EagleImp* and its FPGA extension are freely available at https://github.com/ikmb/eagleimp and https://github.com/ikmb/eagleimp-fpga.

## 1 Introduction

Genotype phasing and imputation have become standard procedures to improve statistical power in *genome-wide association studies* (GWAS) by increasing genome-wide coverage of variants. The algorithmic complexity of phasing and imputation processes implies runtime and memory demands depending on the number of unique target samples and variants (i.e. the input dataset) and the total number of haplotypes and variants in the reference panel. State-of-the-art reference-based phasing and imputation algorithms such as *Eagle2* (Loh *et al.* 2016), *position-based burrows wheeler transform* (PBWT) (Durbin 2014) and *minimac4* (Fuchsberger *et al.* 2014, Das *et al.* 2016) have been efficiently developed with runtimes better than linear scaling over the size of the reference panel. However, for new reference panels with more than one million reference samples (https://digital-strategy.ec.europa.eu/en/policies/1-million-genomes), this would still result in a runtime of weeks, combined with an extremely increasing system memory demand.

Therefore, we recently introduced technical and algorithmic improvements to phasing and imputation in our new software *EagleImp* (Wienbrandt and Ellinghaus 2022). The main advantages of *EagleImp* over the classical two-step approach with *Eagle2* and *PBWT* are the increased computation speed of a factor between 2 and 10 (depending on the applied multiprocessor configuration) while the phasing and imputation quality is at least maintained or even improved.

Here, we demonstrate how to accelerate the complete phasing and imputation process in *EagleImp* even further by a factor of up to 93% by utilizing a field-programmable gate array (FPGA) for the phasing step. In particular, we targeted a *Xilinx Kintex UltraScale KU115* FPGA device on an *Alpha Data ADM-PCIE-8K5* PCIe accelerator card.

Contrary to common *central processing units* (CPUs), the logic circuits in an FPGA are programmable by their own hardware design, such that simple operations, especially Boolean operations, can be implemented directly in the circuit’s logic. By optimizing the device’s resource usage and implementing many operations in a pipeline chain or in parallel, many operations can be performed concurrently, which in turn can make an FPGA extremely fast and keep the power consumption to a minimum (Mencer *et al.* 2020). The developer can flexibly design the FPGA core with arbitrary word sizes and parallel data channels to implement a core algorithm with the goal of a highly optimized use of the resources of the device. In contrast, CPUs lack this flexibility as they process a “program,” which actually is a chain of subsequent commands. Parallelism is introduced only by multiple CPU cores and pipelining for certain commands, but word sizes and data channels are fixed.

In the recent past, FPGAs have proven to be able to accelerate several applications in bioinformatics, especially in the field of epistasis detection (González-Domínguez *et al.* 2015, Kässens *et al.* 2015, Wienbrandt *et al.* 2019). Genotype phasing is again (at least partly) well-suited for FPGAs. In particular, to accelerate the process of genotype phasing and imputation, we target the phasing preliminaries (i.e. the filtering of input data and building the required data structures), which require the majority of the computational resources in the phasing process. They consist of mostly Boolean operations on arbitrary word sizes easily parallelizable over different independent targets and are therefore highly applicable for an FPGA design.

We seamlessly integrated our FPGA extension in the *EagleImp* software available at https://github.com/ikmb/eagleimp. The source code of the FPGA design written in VHDL is available at https://github.com/ikmb/eagleimp-fpga.

The speed advantage allows, e.g. the use of a higher-value parameter selection for phasing with a resulting improvement in the phasing and imputation quality while maintaining the same runtime.

## 2 Methods

Genotype phasing is used as a preliminary step before genotype imputation to increase imputation quality. It describes the process of determining the *phase* of the heterozygous genotypes in a target dataset, i.e. determining on which DNA strand the variant type allele resides for each heterozygous genotype. In *EagleImp*, the phasing step is reference-based and combined with the imputation step in a single application. The software is based on the popular phasing tool *Eagle2* (Loh *et al.* 2016) and the imputation tool *PBWT* (Durbin 2014).

For a normal imputation task, *EagleImp* executes the following steps: (1) The target (genotypes) and the reference files are loaded into memory. (2) Phasing of the target genotypes iterates over all independent target samples and is divided into the *phasing preliminaries* (2a–d) and the final phasing process (2e): (2a) According to the runtime parameter *K*, the *K*-best matching haplotypes for each target are determined and sorted. (2b) The call locations (i.e. heterozygous genotypes) are discovered. (2c) A *condensed reference* is built consisting only of the haplotype information at call sites from the *K*-best haplotype sequences from the reference and the 1-bit information for each segment between call sites whether the haplotype sequence is consistent with the target in that segment or not. Thus, the condensed reference can be regarded as a bit vector with alternating information for call sites and in-between segments. (2d) From the condensed reference a PBWT (Durbin 2014) is created. 2e. The final phasing process consists of a complex algorithm that finds the best matching paths in the PBWT via a *Beam Search*. From these paths, the most probable phase is called. 3. Imputation uses a PBWT data structure of the complete reference to identify *set-maximal matches* with the phased targets in order to calculate the imputed haplotypes. The imputation results are continuously written to a file during this process.

*EagleImp* provides an increased imputation speed of a factor between 2 and 10 when compared to the classical two-step approach using *Eagle2* and *PBWT* in order. On top of that, we observed a positive impact on phasing and imputation quality.

Summarized, the main improvements introduced by *EagleImp* are the following. (i) We have developed a new .qref format for reference data, which significantly improves the reading time of the reference data. (ii) The PBWT data structure of the *condensed reference* required for each target sample is now stored in a compressed binary format with an index similar to the *FM-index* used for a *burrows-wheeler transformation* (BWT) (Ferragina and Manzini 2000). (iii) Haplotype probabilities are no longer stored in a log-based format and a non-normalized scaling factor is used for the haplotype path probabilities. (iv) The imputation of missing genotypes during phasing is obsolete since the subsequent imputation step imputes missing genotypes for shared variants (between target and reference) in the same way as variants that only occur in the reference. (v) Unlike the original *PBWT* tool, *EagleImp* uses multiple threads for genotype imputation, including the use of multiple temporary output files to reduce the input/output (IO) file bottleneck. (vi) We introduced a conversion of genotypes and haplotypes into a compact representation with integer registers and made extensive use of Boolean and bit masking operations as well as processor directives for bit operations (such as *popcount* for counting the set bits in a register) throughout the application. For more details on the algorithm and its improvements, we refer to *EagleImp* (Wienbrandt and Ellinghaus 2022).

Despite our already achieved performance goals, we identified potential for further acceleration using FPGAs. Table 1 shows three of the benchmark datasets we used (Wienbrandt and Ellinghaus 2022), which we will use here for benchmarking later again. We conducted an exemplary profiling of the largest of these datasets (*COVID.Italy*, according to the number of samples), using single-threaded *EagleImp* runs. The profile is presented in Table 2. We observe that between 65% and 85% of the runtime (depending on the runtime parameter *K*) is caused by the phasing part alone (in contrast to the imputation part), and of these between 54% and 79% refer to building the condensed reference (including the selection of the *K*-best haplotypes) with the subsequent creation of the PBWT structure as part of the phasing preliminaries. Although the proportions may vary depending on runtime parameters and the character of the input dataset (e.g. *K*, the number of (heterozygous) call sites and the size of the reference panel), the profile reveals these two main compute-intensive tasks as best candidates for further acceleration, and as most operations in these steps are based on Boolean operations, they are well-suited for outsourcing into an FPGA design.

**Table 1.** Benchmark datasets for quality assurance and runtime measures with FPGA acceleration in *EagleImp-FPGA*.

	Target	#Variants	#Samples	Ancestry
(1)	*HRC.EUR*	619 872	494	European
(2)	*COVID.Spain*	549 696	1792	Spanish
(3)	*COVID.Italy*	559 519	2113	Italian

(1) is artificially generated from the 494 individuals of European ancestry from the *1000 Genomes Project* (The 1000 Genomes Project Consortium 2015), stripped to variants available at Illumina’s *global screening array (GSA)* (Illumina 2020). (2) and (3) are real-world datasets from our COVID-19 GWAS study presented in (Ellinghaus *et al.* 2020). The datasets are the same as used in (Wienbrandt and Ellinghaus 2022) for benchmarking the *EagleImp* software.

**Table 2.** Profiling of the *COVID.Italy* dataset for two different settings of the phasing parameter *K*.

	*K *=* *10 000			*K *=* *32 768		
	Runtime (*s*)	% of total	% of phasing	Runtime (*s*)	% of total	% of phasing
Total	114 602			198 363		
1. Read data	1081	0.94		1029	0.52	
2. Phasing+preliminaries	78 013	68.07		165 798	83.58	
2a. Best haps	1691	1.48	2.17	1799	0.91	1.09
2b. Call locations	255	0.22	0.33	706	0.36	0.43
2c. Condensed reference	16 498	14.40	21.15	52 030	26.23	31.38
2d. Create PBWT	25 613	22.35	32.83	78 551	39.60	47.38
2e. Phasing	33 751	29.45	43.26	32 101	16.18	19.36
3. Imputation	35 490	30.97%		31 518	15.89	

The runs were performed single threaded on the CPU with all chromosomes running in parallel on our benchmark system. Runtimes (in seconds) are accumulated over the chromosomes for each entry.

Therefore, we targeted an Alpha Data ADM-PCIE-8K5 FPGA accelerator card (equipped with a Xilinx Kintex UltraScale KU115 FPGA, two attached 8 GB SODIMM memory modules and a PCI Express Gen3 ×8 connection to the host). In contrast to our first proof-of-concept presented in (Wienbrandt *et al.* 2020), we improved the design significantly. We added an entity that selects the *K*-best haplotypes for each target from the reference to build the *condensed reference* (*K* is a user option and defaults to *K *=* *10 000 in *Eagle2*), and we have implemented the conversion of the condensed reference to the PBWT data structure on the FPGA. (This includes forward and backward PBWT as required for the forward and reverse phasing steps.) Furthermore, matrix transposition for an intermediate conversion to a sample major format for FPGA processing is not required anymore, as the new FPGA design processes the data in native variant-major format, which is the format entirely used in *EagleImp* and in the *variant call format* input files.

**Figure 1.**
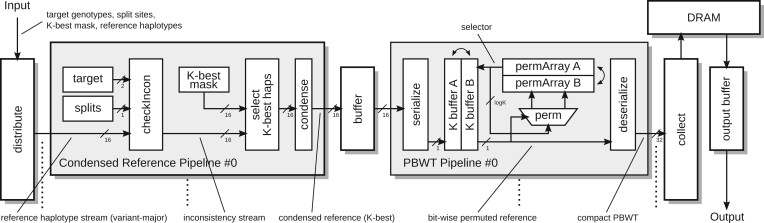
The FPGA pipeline design to create the condensed reference and the PBWT data structure (the most time consuming parts in the phasing process). For simplicity, the figure shows 1-bit processing per clock cycle. In reality, the implemented design processes 2 bits in one clock cycle exploiting dual-port BRAMs and doubling data, address, permutation, multiplexer and output routes. We implemented 32 parallel pipelines on our target FPGA.

We implemented 32 parallel pipelines for concurrent target processing on the FPGA. Each pipeline is divided into two major parts: (i) creation of the condensed reference (*condensed reference pipeline*) and (ii) subsequent generation of the *PBWT* data structure (*PBWT pipeline*). In addition, a preliminary target preparation step and a subsequent data collection step are required on the host system. We illustrated our FPGA design in Fig. 1.

### 2.1 Target preparation

As the FPGA design contains 32 parallel pipelines for concurrent target processing, the host system divides all targets into blocks of 32 target samples for transmission to the FPGA. As in CPU-only phasing, the host determines the call sites and the *K*-best reference haplotypes for each target. The FPGA is then initialized with a number of constants (mainly the size of the targets and the reference panel). The data for each target provided by the host contains the complete genotype information of the targets [encoded as two bitstreams with the first indicating the genotypes that are the homozygous reference type (“is 0”), and the second indicating the genotypes that are homozygous alternative type (“is 2”)], the call sites (encoded as a bitstream with one bit for each site where a set bit indicates a call site), and a bit mask for the *K*-best haplotypes (i.e. one bit for each haplotype with a set bit indicating that this haplotype is to be used in the analysis). The FPGA distributes the target data from the host to the FPGA pipelines in a simple round-robin scheme, whereby a block of targets has to be processed completely before the next block can be initialized.

### 2.2 Condensed reference pipeline

After the initialization of each pipeline with the target data, the host streams the reference data to the FPGA in variant-major format, i.e. for each variant the haplotype data of all samples is provided. The reference data has to be streamed completely and repeated for each block of targets. The FPGA processes one variant after the other. The reference data is broadcast to all pipelines in parallel with 16 bits of haplotype information per clock cycle. The *checkIncon* unit produces an *inconsistency stream* by checking the consistency of the 16 presented haplotypes to the current genotype according to the following equation [is0 and is2 indicating if the current genotype is homozygous reference 0 or homozygous variant (2), hap is the haplotype data encoded as 0 for reference and 1 for variant type]:

(1) inc=(is0ANDhap)OR(is2AND NOThap)

An exception is made for variants that are marked as a call site which is at least true for all heterozygous sites. The haplotype data of these variants is forwarded unchanged.

The subsequent unit selects the *K-best haplotypes* from the incoming stream. For each incoming 16-bit word, the corresponding 16-bit mask is used to remove the unneeded haplotypes. As for the follow-up processes it is required to reduce the stream of *n* bits for each variant (one bit for each reference haplotype) to only *K* bits, the unit reduces the incoming 16-bit words to words of a size that matches the number of set bits of the current mask. This is achieved by using a routing similar to an *odd-even transposition sort* sorting network, where the mask is sorted and the data routed alongside the 1 bits of the mask. The resulting smaller words are then collected until full 16-bit words are available, which are provided to the *condense* unit afterwards.

The *condense* unit collects the information for all variants that are not marked as call sites (which is the current inconsistency information) in a local BRAM that is able to store *K* bits. The data for all subsequent variants that are not call sites are merged with the currently stored data by a simple logical OR operation in order to generate the information for the current inconsistency segment in the condensed reference. This process continues until a variant is marked as a call site which triggers the release of the data stored in the local BRAM (which is the complete inconsistency information for the current segment) and the incoming data from the call site to be stored. The next variant triggers the release of the just-stored data for the call site while the incoming data is now stored in the BRAM.

The outgoing condensed reference is then buffered before being converted to a PBWT structure in the *PBWT pipeline* in the next step. The buffer is able to block the preliminary processes to ensure no data loss in the case the subsequent pipeline has not finished with the preliminary variant yet. Furthermore, it ensures the correct crossing of clock domains as the creation of the condensed reference runs with a different clock frequency than the following PBWT creation.

### 2.3 PBWT pipeline

A PBWT (Durbin 2014) is a per-site permutation such that the original haplotypes appear sorted when read in the permuted order backward starting from that site. So, the required PBWT structure for the phasing process is a per-site permutation from the so-far created condensed reference.

The design of the PBWT pipeline presented here is the exact hardware implementation of the algorithm for compact PBWT creation provided in the following Listing 1.

Listing 1. PBWT creation from *N* Boolean sequences of length *M*.

**input** haps[M][N]          *# haplotype data*

**input** cnt0[M]         *# number of zeros for each site*

**output** pbwt[M][N]           *# PBWT permuted haplotype data*

**define** a[N]           *# current permutation array*

**define** b[N]      *# next permutation array*

*# initial permutation is the identity*

**for** n in [0.N-1] **do**

  a[n] = n

**done**

*# loop over all sites*

**for** m in [0.M-1] **do**

 p0 = 0

 p1 = cnt0[m]

 **for** n in [0.M-1] **do**

  **if** haps[m][a[n]] == 0 **then**

   *# current entry in permutation order is 0*

   b[p0] = a[n]

   pbwt[m][n] = 0

   p0 = p0 + 1

  **else**

   *# current entry in permutation order is 1*

   b[p1] = a[n]

   pbwt[m][n] = 1

   p1 = p1 + 1

  **fi**

 **done**

 **swap** a, b

**done**

To generate the permuted haplotypes, the PBWT unit contains two haplotype buffers for storing up to *K* bit of haplotype data each, and two permutation arrays that can store up to *K* permutation indices (of size ${\;\text{log}\;}_{2}K$). The buffers require access to an arbitrary stored haplotype bit preferably in a single clock cycle, which is implemented in the FPGA’s local BRAM. (In fact, the data is available in the next clock cycle after the required address has been provided, but the process is pipelined such that each clock cycle a different address can be provided with the output appearing with one cycle delay.)

The PBWT generation process is conducted as follows. First, the incoming condensed reference data (in 16-bit words) is serialized to a bitwise stream. The data for the first site is simply stored in the first haplotype buffer at the beginning, and the first permutation array is initialized with the identity function (i.e. no permutation will be made). Meanwhile, for each site, the number of zero bits (*cnt0*) is determined from the serialized stream. For the following sites, the bitstream is stored in the second haplotype buffer while the first buffer is read according to the permutation stored in the first permutation array. In detail, the permutation indices are read subsequently and provided as an address to the haplotype buffer. The resulting data stream is now in the required permuted order for the compact PBWT representation and is provided to a deserialization unit that prepares 32-bit output words.

To generate the next permutation, the second permutation array is implemented as two queues: the first one starting at the beginning of the array for storing the permutation indices that addressed an unset 0 bit in the haplotype buffer, and the second one starting at *cnt0* for storing the indices that addressed a set (1) bit. Each current permutation index is then written to the second (new) permutation array with its destination dependent on the corresponding haplotype bit: if the bit is zero the index is written to the first queue, if it is one it is written to the second queue. After the last data from the current site, all arrays are switched before the next site, i.e. the first haplotype buffer becomes the second and vice versa, and the first permutation array becomes the second and vice versa.

The generation of the compact PBWT is the major bottleneck of our FPGA design as the complete condensed reference needs to be processed bit by bit. We were able to increase the data throughput by processing two bits of the condensed reference in one clock cycle. For this, we used the dual-port feature of the FPGA’s BRAM units. In detail, two permutation indices are read from the first permutation array in each clock cycle. Both indices request a data bit from the haplotype buffer from different addresses in the same clock cycle, which is only possible with the dual-port feature. The resulting two data bits generate four possibilities of writing the two indices to the second permutation array: (i) both indices get into the first queue, (ii) the first index gets into the first queue and the second in the second queue, (iii) the first gets into the second queue and the second gets into the first queue, and (iv) both indices get into the second queue. Again, this is only possible using the BRAM’s dual-port feature.

For a maximum throughput, the design’s clock frequency is a major factor. We are able to operate the PBWT creation at an increased frequency of 266 MHz while the rest of the pipeline operates at 114 MHz. This is a factor of 2.33 times faster but while the PBWT unit processes only 2 bits per clock cycle and the condensed reference is provided with 16 bits per clock cycle, we can predict the throughput of the PBWT unit to be $\frac{16}{2}\times \frac{114}{266}=3.43$ times slower than the *maximum* throughput of the condensed reference unit. However, because the condensed reference unit provides its output at a different (in general lower) speed than the input of the raw data is processed (as only call sites to generate sites in the condensed reference and the reference is reduced to the *K*-best haplotypes only), the bottleneck is reduced or even eliminated in most cases making the FPGA work at a maximum efficiency.

### 2.4 Data collection

Before sending the PBWT data to the host system it is buffered in the attached DRAM. The output words (32 bits) from each deserialization unit are collected to form 512-bit words according to the size of the DRAM data port. Due to the processing width of 2 bits at a frequency of 266 MHz the PBWT unit can generate a 512-bit word every 256 clock cycles, i.e. every 110 clock cycles in the 114 MHz clock domain. This leaves enough space for the DRAM to handle the output of the 32 parallel pipelines without generating another bottleneck. We store the compact PBWT data of each target ordered in subsequent memory areas in the DRAM, each area designated to one pipeline. The size of the memory area depends on the number of expected DRAM words for the designated target PBWT and is simply achieved by adding an address offset to each pipeline corresponding to the number of expected DRAM words for all preliminary pipelines.

As our *Alpha Data* FPGA accelerator board is equipped with two DRAM modules, we implemented two DRAM buffers as well. While one buffer is filled by the processing pipelines for the current target block, the other buffer is read and the data from the previous target block is transferred to the host system simultaneously.

The host system generates the index fields required for fast access to the PBWT data on the fly while copying the received data from the transfer buffer to host memory. The index is simply implemented as a 32-bit integer attached to each 32-bit block of compact PBWT data. The integer value is the number of zero bits from the beginning of the current site up to the current block (inclusive). We decided against the implementation of the FPGA as this doubles the amount of data to be transferred to the host and we do not want to risk becoming the bottleneck of the design. Furthermore, this process is very efficiently implemented on the host by using the *popcount* processor directive that determines the number of set bits in a 32-bit word in a single processor command.

### 2.5 Phasing process

The second main part of the phasing step of *EagleImp* is the phasing process. The created *PBWT* data structure is used to find the best matching paths in the reference to the target via a *Beam Search*. These paths are then used to determine the phase probabilities of the heterozygous genotypes in the target, and thus, do the final phase calling. In *EagleImp-FPGA* we have not modified this part in the phasing step or the subsequent imputation step. Instead, the outsourced phasing preliminaries are neatly integrated into the *EagleImp* software such that all routines are used as before in *EagleImp* without FPGA acceleration.

## 3 Results

We previously showed that *EagleImp* delivers at least the same or better phasing and imputation quality (in terms of phasing switch error rate and imputation genotype error rate) compared to the tools *Eagle2* (Loh *et al.* 2016) and *PBWT* (Durbin 2014), with a speed advantage of a factor between 2 and 10 (depending on the applied multiprocessor configuration) (Wienbrandt and Ellinghaus 2022). For common variants examined in typical GWAS studies, we were also able to show that *EagleImp* had the same or higher imputation accuracy (in terms of imputation accuracy *r*^2^) than the *Sanger Imputation Service* (Wellcome Sanger Institute 2022), the *Michigan Imputation Server* (US National Institutes of Health 2024) and the *TOPMed Imputation Server* (National Heart, Lung, and Blood Institute 2024), despite larger (not publicly available) reference panels.

**Figure 2.**
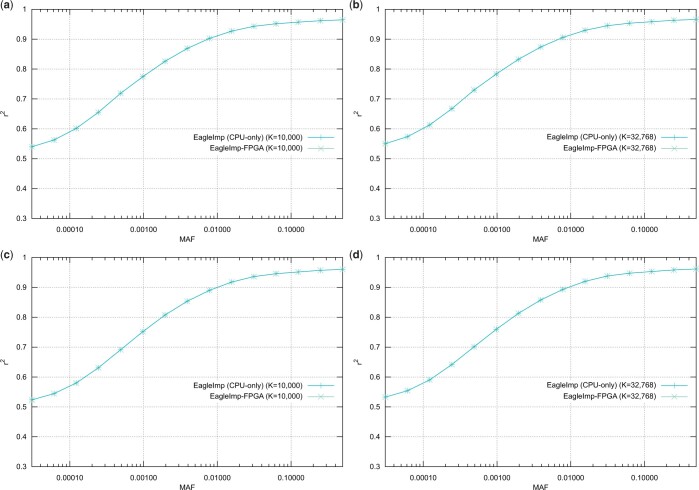
Imputation accuracy *r*^2^ stratified by minor allele frequency (MAF) for *K *=* *10 000 and *K *=* *32 768 for two real-world datasets *COVID.Spain* (a–b) and *COVID.Italy* (c–d) imputed using the EGA release of the HRC1.1 reference panel. The runs with and without FPGA acceleration do not show any difference demonstrating that there is no impact on imputation quality when using FPGA acceleration for the preliminary phasing step in *EagleImp*.

For our presented FPGA design, which we implemented on the beforementioned *Alpha Data* FPGA accelerator with 32 parallel pipelines, we conducted quality assurance benchmarks depending on two different values of the phasing parameter *K* (to select the K-best haplotypes from the reference for phasing) by repeating the benchmarks in (Wienbrandt and Ellinghaus 2022) for the three datasets *HRC.EUR*, *COVID.Spain* and *COVID.Italy* (see Table 1) with FPGA acceleration enabled. We confirmed for the *HRC.EUR* dataset (comprising 494 European samples) the same phasing switch error rate of 0.00470 for *K *=* *10 000 and 0.00434 for *K *=* *32 768 when phased against the EGA release of the *HRC1.1* reference panel. The imputation genotype error rates were confirmed with 0.00265 for *K *=* *10 000 and 0.00254 for *K *=* *32 768, respectively (see also Fig. 3 in Wienbrandt and Ellinghaus 2022). Consequently, the imputation accuracy *r*^2^ shows the same behavior as demonstrated by determining the *r*^2^ values stratified by minor allele frequency for *K *=* *10 000 and *K *=* *32 768 for the real-world datasets *COVID.Spain* and *COVID.Italy* (from Ellinghaus *et al.* 2020, comprising 1792 Spanish and 2113 Italian samples, respectively, see Table 1) with the resulting diagrams showing no difference in phasing and imputation quality and accuracy between CPU-only and FPGA-accelerated runs for the same *K* (see Fig. 2a–d).

To determine the speed advantage of the FPGA implementation over the CPU-only implementation, we performed a runtime analysis of the same three benchmark datasets and the same *K* values from our quality assurance benchmarks above. We used the same computing system as for the runtime benchmarks in (Wienbrandt and Ellinghaus 2022) (equipped with two Intel Xeon E5-2667 v4 CPUs, each with 8 cores running at 3.2 GHz resulting in 32 available system threads, and 256 GB of DDR4 RAM). The CPU-only runs were performed with *EagleImp* in a 2×4×8 configuration (i.e. eight worker processes with eight threads each are started simultaneously, while every two workers share a lock for multiple exclusion on CPU resources). This is the fastest multiprocessor configuration for *EagleImp* as reported in Wienbrandt and Ellinghaus (2022). For *EagleImp-FPGA*, we used a multiprocessor configuration of eight worker processes with 16 threads each, while each two workers share a lock for multiple exclusion on CPU resources (hereafter referred to as 2 × 4 × 16 configuration). This can be done without overloading the system threads because all worker processes have to share the single FPGA resource via multiple exclusion as well.

**Figure 3.**
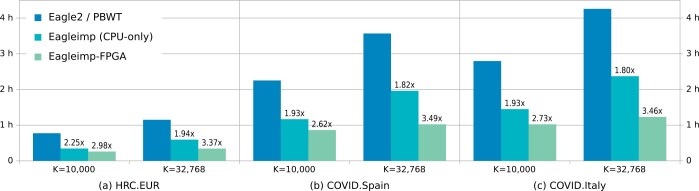
Phasing and imputation wall-clock runtimes for the three benchmark datasets from Table 1: (a) *HRC.EUR* (494 European samples with 619 872 variants), (b) *COVID.Spain* (1792 Spanish samples with 549 696 variants) and (c) *COVID.Italy* (2113 Italian samples with 559 519 variants) phased and imputed with (i) the combination of *Eagle2* (phasing) and *PBWT* (imputation), (ii) *EagleImp* (CPU-only), and (iii) *EagleImp-FPGA* using a Xilinx UltraScale KU115 FPGA accelerator. Phasing and imputation was conducted using the HRC1.1 EGA release reference panel and two different values of the phasing parameter *K*. Speedup factors on top of each bar are relative to the corresponding *Eagle2/PBWT* run.

For the *HRC.EUR* dataset and *K *=* *10 000, the FPGA run took 15 min and 32 s, and for *K *=* *32 768, the runtime was 20 min and 25 s. This is an acceleration of 32% and 74%, respectively, when compared to the CPU-only runs. The larger real-world GWAS datasets *COVID.Spain* and *COVID.Italy* showed an even greater speedup for FPGA-accelerated *EagleImp* of 35% and 42% for *K *=* *10 000 and 92% and 93% for *K *=* *32 768 when compared to the CPU-only runs. When compared to the original tools *Eagle2* and *PBWT*, this corresponds to an increase of the speedup factors of *EagleImp* from 1.94, 1.82 and 1.80 to 3.37, 3.46 and 3.49, respectively, for the *HRC.EUR*, *COVID.Spain* and *COVID.Italy* (*K *=* *32 768) due to FPGA acceleration (see Fig. 3). Note that *Eagle2/PBWT* was also run in the fastest multiprocessor configuration with eight workers running in parallel with each worker utilizing four threads.

**Figure 4.**
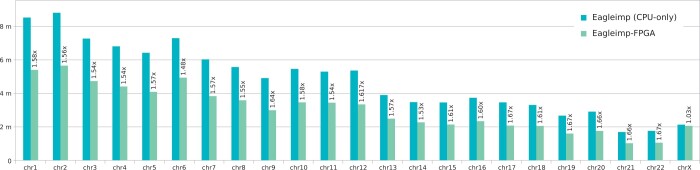
Phasing-only chromosome-wise wall-clock runtimes for the *COVID.Italy* dataset (2113 Italian samples with 559 519 variants) phased against the HRC1.1 EGA release reference panel with phasing parameter *K *=* *32 768 using (i) *EagleImp* (CPU-only) and (ii) *EagleImp-FPGA* using a Xilinx UltraScale KU115 FPGA accelerator. For each run all available 32 system threads were used.

As the FPGA only accelerates a part of the phasing step in the complete imputation process, we conducted a further benchmark of the *COVID.Italy* dataset with *K *=* *32 768 where we measured the runtimes of each chromosome file for the phasing step only, once processed in a CPU-only run using all available 32 system threads, and once processed with FPGA acceleration. The results are illustrated in Fig. 4 and show an FPGA speedup for chromosomes 1–22 ranging between 48% and 67% for phasing alone. As chromosome X receives special treatment due to haploid samples, it is an outlier with almost no acceleration (only 3%).

## 4 Discussion

We demonstrated that FPGA acceleration in *EagleImp* provides a speed advantage over the CPU-only run of up to 93%, with no loss in quality and accuracy. In comparison to classical *Eagle2/PBWT* phasing and imputation, even when conducted with a fast multiprocessor configuration (using eight workers with four threads each), *EagleImp* with FPGA extension can be approximately 3.5 times faster, hence reducing the runtime for our *COVID.Spain* dataset from 3.5 h to only 1 h and for the *COVID.Italy* dataset from 4 h and 15 min to only 1 h and 14 min.

This speedup is gained from two main acceleration steps. Firstly, algorithmic and data IO improvements introduced by *EagleImp* in addition to the possibility to place a lock on CPU resources for the compute-intensive (multithreaded) tasks make it possible to use the CPU resources more efficiently. Note that for a more efficient CPU utilization, we also started *Eagle2* phasing with 8 concurrent jobs and 4 threads each (referred to as 8×4 multiprocessor configuration), and *PBWT* with all jobs concurrently (as it does not support multithreading). Still, *EagleImp* (without accelerator) was approximately 2 times faster. (For more information on benchmarks with different multiprocessor configurations, see Wienbrandt and Ellinghaus 2022 again. The 8 × 4 configuration is reported as the fastest and used for comparison here.)

Secondly, further acceleration was achieved by the presented FPGA implementation. *EagleImp-FPGA* outsources the phasing preliminaries, a part of the complete phasing step, to an FPGA resource. Note that not the complete phasing step could be outsourced, and therefore, the total runtime of this process could not be smaller than the runtime of the part that we were not able to implement on the FPGA. In order to further analyze this, we conducted chromosome-wise phasing without running several jobs in parallel but using all available 32 system threads for the phasing step. We observed FPGA acceleration factors mostly between 48% and 67% (with the exception of one outlier for chromosome X). Considering that only the phasing preliminaries were accelerated by the FPGA, which presents approximately only 50% of the complete step, we regard this as a satisfying result.

In addition, our FPGA-based phasing shows no loss of quality compared to the CPU-only version, but, due to the speed advantage, allows the choice of a higher value for the *K* parameter for phasing, which further reduces the phasing switch error rate and the genotype imputation error rate. This also increases the imputation *r*^2^ which provides higher-quality association results in subsequent GWAS. (A correlation of an increasing *K* with a higher imputation quality was also demonstrated in Wienbrandt and Ellinghaus 2022.)

For further development, we focus on the issue that input data containing haploid samples is not efficiently processed by our FPGA extension yet (see the processing of the chromosome X file in the chromosome-wise benchmark of the *COVID.Italy* dataset that led to a speedup of only 3%). The idea is to filter haploid samples from the phasing process beforehand, and not to handle them as homozygous diploid as it is implemented now.

## Data Availability

*EagleImp* software: https://github.com/ikmb/eagleimp. *EagleImp-FPGA* design sources: https://github.com/ikmb/eagleimp-fpga.
